# Association Between the Highest Lactate Level on the First Postoperative Day and Postoperative Delirium in Cardiac Surgery Patients

**DOI:** 10.1111/cns.70380

**Published:** 2025-04-22

**Authors:** Ran An, Xie Wu, Dongyun Bie, Jie Ding, Yinan Li, Yuan Jia, Su Yuan, Fuxia Yan

**Affiliations:** ^1^ Department of Anesthesiology, Fuwai Hospital, National Center of Cardiovascular Diseases Chinese Academy of Medical Sciences and Peking Union Medical College Beijing China

**Keywords:** cardiac surgery, postoperative delirium, risk factor, the highest lactate level

## Abstract

**Aims:**

The study aimed to determine the correlation between the maximum lactate on the first postoperative day and the incidence of postoperative delirium (POD) in patients after cardiac surgery.

**Methods:**

The data of cardiac surgery patients were extracted from the Medical Information Mart for Intensive Care IV database. The cut‐off value for the first postoperative day maximum lactate was determined, and all patients were categorized into two groups according to the cut‐off value. Propensity score matching (PSM) was applied between the two groups, and the difference in the incidence of POD was analyzed. Then, we employed univariate logistic regression, multivariate logistic regression, PSM, and inverse probability of treatment weighting (IPTW) models to examine the relationship between the first postoperative day lactate levels and POD.

**Results:**

Among the 4856 patients enrolled, there was a significant difference in lactate‐max on the first postoperative day between patients without POD and patients with POD (median 2.5 vs. 3.1, *p* < 0.001). The cut‐off value of lactate‐max was 2.85 mmol/L. For the two groups after PSM, the incidence of POD in the lactate‐max ≥ 2.85 mmol/L group was significantly elevated (19.2% vs. 15.9%, *p* = 0.029). The elevated lactate‐max on the first postoperative day was substantially associated with an increased risk of POD in univariate and multivariate logistic regression analyses, PSM, and IPTW models.

**Conclusions:**

The results demonstrated that the first postoperative day lactate‐max was correlated with the risk of POD in patients undergoing cardiac surgery, with the POD risk increasing significantly in patients with a lactate‐max ≥ 2.85 mmol/L on the first postoperative day.

## Graphical abstract

This large‐sample retrospective cohort study demonstrated that the first postoperative day lactate‐max was associated with the risk of POD in patients undergoing cardiac surgery, with the POD risk increasing significantly in patients with a lactate‐max ≥ 2.85mmol/L on the first postoperative day.

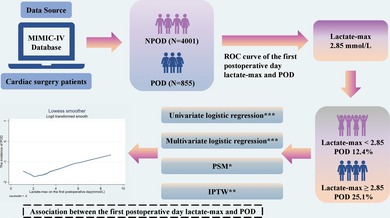

## 1 Introduction

Delirium is a prevalent yet severe neuropsychiatric syndrome characterized by fluctuating cognitive impairment [1]. Postoperative delirium (POD) frequently occurs in patients undergoing cardiac surgery during their intensive care unit (ICU) stay, with incidence ranging from 3% to 78% [2, 3]. It has been found that POD is linked to adverse outcomes, including prolonged hospitalization, persistent cognitive impairment, and increased mortality [4, 5, 6, 7]. Extensive research has been conducted on POD prevention, leading to the establishment of several effective preventive strategies [8, 9]. Therefore, the early identification of high‐risk populations and the prevention of POD are crucial.

Lactate is a widely used clinical biomarker for microcirculation, indicative of tissue perfusion [10]. Recently, there has been growing attention to the impact of perioperative serum lactate levels on the short‐term adverse outcomes of surgical patients [11, 12, 13]. Previous research has demonstrated that elevated perioperative lactate was a significant predictor of in‐hospital mortality and 30‐day or 90‐day mortality in patients receiving gastrointestinal surgery [14], orthopedic surgery [15], hepatectomy [16], and other procedures. Ludhmila et al. proved that hyperlactatemia at 6 h after cardiac surgery was a risk factor for adverse outcomes, including postoperative 30‐day all‐cause mortality and severe complications such as cardiogenic shock, acute respiratory distress syndrome, acute kidney injury, and renal replacement therapy during the postoperative hospital stay [12]. Nevertheless, limited research has focused on the impact of perioperative serum lactate levels on adverse neurological outcomes such as POD, particularly in patients undergoing cardiac surgery.

Consequently, we conducted this study to further assess the association between the peak lactate levels on the first postoperative day and POD in patients undergoing cardiac surgery. The primary hypothesis of the study was that, among cardiac surgery patients, those with elevated lactate‐max on the first postoperative day had a higher risk of POD.

## 2 Methods

### 2.1 Data Source

The study data were extracted from a publicly available database, the Medical Information Mart for Intensive Care IV (MIMIC‐IV) database (version 2.2). This database contains comprehensive medical information of ICU patients admitted to Beth Israel Deaconess Medical Center between 2008 and 2019 [17]. Due to the de‐identified data in this database, it's not necessary to obtain informed consent from patients for the study. One of our authors has completed the requisite training to utilize the database and was approved to access and obtain the data from the MIMIC‐IV database (Record ID: 55754432). This study followed the recommendations of Strengthening the Reporting of Observational Studies in Epidemiology (STROBE) guidelines.

### 2.2 Patient Selection

**FIGURE 1:**
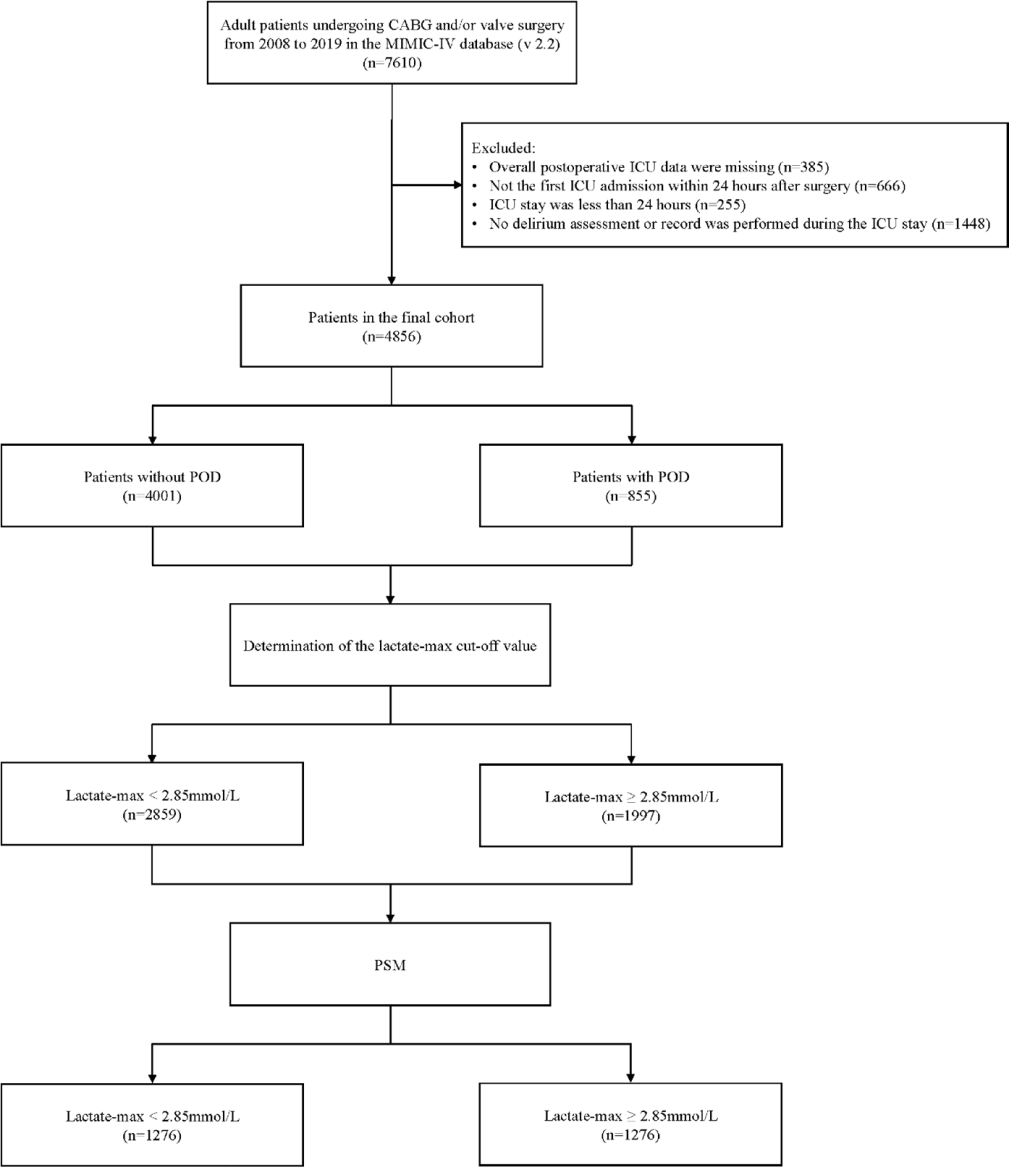
Flowchart for patient selection. CABG, coronary artery bypass grafting; MIMIC‐IV, Medical Information Mart for Intnsive Care IV; ICU, intensive care unit; POD, postoperative delirium; PSM, propensity score matching.

Patients were included if they met the following criteria: (1) underwent coronary artery bypass grafting (CABG) and/or valve surgery; (2) aged ≥ 18 years; (3) were admitted to the ICU within 24 h after surgery. We excluded the following patients: (1) those with missing data, such as absent lactate levels on the first postoperative day; (2) individuals whose postoperative ICU stay was less than 24 h; (3) patients who were not evaluated or documented for POD during their ICU stay. International Classification of Diseases (ICD)‐9 and ICD‐10 codes were used to identify cardiac surgery in the “procedures_icd” table of the MIMIC‐IV database, as detailed in Table S1. If patients underwent several cardiac surgeries, only the first procedure was considered in the analysis. Upon applying all the above inclusion and exclusion criteria, we ultimately identified 4856 eligible patients for subsequent analyses. Figure 1 presents the details of the patient screening process.

### 2.3 Data Extraction

The data were extracted from the MIMIC‐IV database using Postgres Structured Query Language. The extracted data comprised: (1) baseline characteristics; (2) concomitant disorders; (3) preoperative laboratory data; (4) severity of illness after ICU admission; (5) postoperative laboratory data on the first day after surgery; (6) special therapy within the first 2 days after surgery. The data of patients undergoing cardiac surgery were collected from the “d_icd_procedures” and “procedures_icd” tables in the “mimic_hosp” module of the MIMIC‐IV database, using the ICD codes listed in Table S1. The surgery type and time were extracted as well. Basic patient information, such as admission type, race, age, gender, and body mass index, was then extracted from the “admissions”, “patients”, and “omr” tables. Comorbidities were extracted from the “diagnoses_icd” and “d_icd_diagnoses” tables. Laboratory examination results were obtained from the “labevents” and “d_labitems” tables. ICU stay duration was extracted from the “icustays” table in the “mimic_icu” module. Detailed information on specific treatments and medications administered during the ICU stay was extracted from the “procedureevents” and “ingredientevents” tables. The delirium assessment results during the ICU stay were extracted from the “d_items” and “chartevents” tables.

The lactate‐max on the first postoperative day was defined as the highest value of all lactate measurements taken within the first 24 h following surgery. The primary outcome of POD was defined as delirium occurring during the postoperative ICU period. For patients included in the MIMIC‐IV database, delirium was screened using the confusion assessment method for the intensive care unit (CAM‐ICU) and diagnosed according to the Diagnostic and Statistical Manuals of Mental Disorders [18]. Specifically, patients were first assessed for sedation status with the Richmond Agitation and Sedation Scale (RASS), and delirium was evaluated when the RASS score ≥ −3. The CAM‐ICU comprises four components: (1) acute mental status change (MIMIC‐IV itemid 228,337, 228,300, and 229,326); (2) inattention (MIMIC‐IV itemid 228,301, 228,336, and 229,325); (3) disorganized thinking (MIMIC‐IV itemid 228,335, 228,303, and 229,324); (4) altered level of consciousness (MIMIC‐IV itemid 228,334). Delirium is diagnosed when traits 1 and 2, along with either 3 or 4, are present. The secondary outcomes included the length of ICU stay, length of hospital stay, in‐hospital mortality, and postoperative 30‐day mortality.

### 2.4 Covariates and Propensity Score Matching

Based on previous literature, clinical experience, and data availability, we identified variables that potentially affect the risk of delirium as covariates, including baseline patient characteristics, comorbidities, preoperative laboratory test results, severity of illness after ICU admission, laboratory test results on the first day after surgery, and special treatment administered within 2 days after surgery (Table 2).

To ensure the robustness and stability of the study results, we adopted propensity score matching (PSM). Prior to implementing PSM, we addressed the issue of missing data. The proportion of missing data for all variables in this study did not exceed 10%. Missing values were inferred using the assumption of missing at random, and multiple imputation by chained equations was employed to estimate and impute the missing values precisely [19]. Based on the aforementioned covariates, a propensity‐scoring model was developed. We employed a logistic regression model to estimate the propensity score for each patient and conducted the hypothesis testing during the model construction process to ensure the stability and accuracy of the model. To achieve the best match, we adopted the nearest neighbor matching combined with caliper matching at a 1:1 matching ratio. The initial caliper width was set to 0.2 times the standard deviation of the propensity score. After iterative adjustment, we determined the optimal caliper value to be 0.02, ensuring the best matching quality without reducing the sample size after matching. The equilibrium after PSM was evaluated using the standardized means difference (SMD), with SMD < 0.05 considered indicative of good balance [20].

### 2.5 Statistical Analysis

All analyses were conducted using Stata Software STATA 15.0 (StataCorp Stata MP 15.0, United States), and a two‐tailed *p* < 0.05 was considered statistically significant.

#### 2.5.1 Data Distribution and Group Comparison

The Shapiro–Wilk test was employed to evaluate the data distribution of all continuous variables, revealing that all were non‐normally distributed. Consequently, the continuous variables were presented as median (interquartile range [IQR]) and were compared by the Mann–Whitney *U* test or Wilcoxon rank‐sum test. The categorical variables were represented as counts (percentages) and compared using the chi‐square test.

#### 2.5.2 Determination of the Cut‐Off Value Using the Receiver Operating Characteristic Curve

To determine the cut‐off value of lactate‐max, a receiver operating characteristic (ROC) curve was constructed to analyze the relationship between the maximum lactate levels on the first postoperative day and the incidence of POD. The cut‐off value for maximum lactate was determined by maximizing the correct classification rate [21]. Based on the cut‐off value, all subjects were divided into two groups, and lactate‐max was converted from a continuous variable to a binary variable for subsequent analysis.

#### 2.5.3 Logistic Regression Analyses

Univariate and multivariate logistic regression analyses were employed to evaluate the correlation between the lactate‐max on the first postoperative day and the incidence of POD, and the odds ratio (OR) with a 95% confidence interval (CI) was calculated [22]. Variables with *p* < 0.05 identified in univariate logistic regression analysis and recognized as clinically significant were incorporated into the multivariate logistic regression model using stepwise regression to minimize collinearity while retaining essential variables. Collinearity was assessed using the variance inflation factor (VIF), with VIF < 5 indicative of no significant collinearity [23]. The variables included in the final multivariate logistic regression model were presented in detail in Table S2.

Additionally, multivariate logistic regression analysis was performed within PSM and inverse probability of treatment weighting (IPTW) models [20, 24]. For the IPTW model, a logistic regression model was employed to construct a propensity score model for the aforementioned covariates and calculate inverse probability weights, based on which a pseudo‐population with balancing confounding factors between the lactate‐max < 2.85 mmol/L and lactate‐max ≥ 2.85 mmol/L groups was generated. SMD was utilized to assess the equilibrium between the two groups in the IPTW model, and it is widely recognized that an SMD < 0.05 indicates good balance [24].

#### 2.5.4 Locally Weighted Scatterplot Smoothing

Locally weighted scatter plot smoothing (Lowess) was utilized to illustrate the relationship between the lactate‐max and the incidence of POD [25]. This non‐parametric approach can effectively visualize the potential non‐linear association between lactate‐max and POD, and the smoothed curve offered insights into the trend of the incidence of POD with different lactate‐max levels.

#### 2.5.5 Subgroup Analysis

To assess whether the results were robust, we conducted subgroup analyses in the PSM model to assess potential interactions between the maximum lactate level on the first postoperative day and various stratification variables, including age, gender, admission type, history of cerebrovascular disease, early postoperative administration of midazolam, and duration of mechanical ventilation.

## 3 Results

### 3.1 Clinical Characteristics

A total of 4856 patients were included in the final analysis of the study. Figure 1 illustrates the screening process for patients. As shown in Table 1, the median age of the enrolled patients was 68 years (IQR 60–75 years), and 27.7% were female. 2881(59.3%) patients underwent CABG, 1251(25.8%) patients underwent valve surgery, 724(14.9%) patients underwent combined surgery. The patients were categorized into two groups based on the occurrence of delirium during their ICU stay, with 4001 (82.4%) patients without POD and 855 (17.6%) patients with POD. The baseline and clinical data of the two groups were compared, encompassing baseline characteristics, concomitant disorders, preoperative laboratory data, the severity of illness after ICU admission, postoperative laboratory data on the first day after surgery, and special treatment in the first 2 days after surgery. Overall, patients with POD were older, exhibited a higher proportion of combined surgery, and presented with more preoperative comorbidities. The results suggested a significant difference in lactate‐max on the first postoperative day between patients without POD and patients with POD (median 2.5 vs. 3.1, *p* < 0.001).

**TABLE 1:** Baseline characteristics and clinical data of patients without POD and patients with POD.

	Total (*n* = 4856)	NPOD (*n* = 4001)	POD (*n* = 855)	*p*
Baseline characteristics				
Age, years	68 (60–75)	67 (59–75)	71 (62–77)	< 0.001
Gender				
Male	3512 (72.3%)	2942 (73.5%)	570 (66.7%)	< 0.001
Female	1344 (27.7%)	1059 (26.5%)	285 (33.3%)	
Ethnicity				
White	3284 (67.6%)	2705 (67.6%)	579 (67.7%)	0.190
African American	163 (3.4%)	126 (3.2%)	37 (4.3%)	
Unknown/Others	1409 (29.0%)	1170 (29.2%)	239 (28.0%)	
BMI, kg/m^2^	29.0 (25.7–32.9)	29.1 (25.8–33.0)	28.7 (25.0–32.9)	0.020
Admission type				
Elective	2049 (42.2%)	1754 (43.8%)	295 (34.5%)	< 0.001
Non‐elective	2807 (57.8%)	2247 (56.2%)	560 (65.5%)	
Surgery type				
CABG only	2881 (59.3%)	2476 (61.9%)	405 (47.4%)	< 0.001
Valve only	1251 (25.8%)	1004 (25.1%)	247 (28.9%)	
Combined (CABG + valve)	724 (14.9%)	521 (13.0%)	203 (23.7%)	
Concomitant disorders				
Congestive heart failure	1359 (28.0%)	998 (24.9%)	361 (42.2%)	< 0.001
Hypertension	2816 (58.0%)	2394 (59.8%)	422 (49.4%)	< 0.001
Pulmonary hypertension	217 (4.5%)	153 (3.8%)	64 (7.4%)	< 0.001
Chronic pulmonary disease	993 (20.5%)	785 (19.6%)	208 (24.3%)	0.002
Peripheral vascular disease	709 (14.6%)	525 (13.1%)	184 (21.5%)	< 0.001
Diabetes	1832 (37.7%)	1455 (36.4%)	377 (44.1%)	< 0.001
Chronic liver disease	197 (4.1%)	150 (3.8%)	47 (5.5%)	0.019
Chronic kidney disease	885 (18.2%)	648 (16.2%)	237 (27.7%)	< 0.001
Cerebrovascular disease	486 (10.0%)	357 (8.9%)	129 (15.1%)	< 0.001
Dementia	26 (0.5%)	16 (0.4%)	10 (1.2%)	0.005
Malignancy	132 (2.7%)	109 (2.7%)	23 (2.7%)	0.955
Alcohol abuse	62 (1.3%)	47 (1.2%)	15 (1.8%)	0.171
Mental disorder				
Anxiety	584 (12.0%)	463 (11.6%)	121 (14.2%)	0.035
Depression	569 (11.7%)	446 (11.2%)	123 (14.4%)	0.008
Preoperative laboratory data				
Hemoglobin, g/dL	12.2 (10.6–13.6)	12.4 (10.7–13.7)	11.4 (9.8–13)	< 0.001
WBC, 10^9^/L	9.2 (7.0–12.9)	9.2 (7.0–12.8)	9.5 (7.2–13.2)	0.050
Platelet, 10^9^/L	170 (130–217)	171 (131–217)	166 (124–217)	0.068
ALT, U/L	22 (15–32)	22 (16–32)	21 (15–33)	0.224
AST, U/L	24 (19–35)	24 (19–33)	27 (20–47)	< 0.001
Albumin, mg/dL	4.1 (3.7–4.4)	4.1 (3.8–4.4)	4.0 (3.6–4.3)	< 0.001
Glucose, mg/dL	112 (96–145)	111 (95–144)	114 (97–150)	0.027
Serum creatinine, mg/dL	0.8 (0.6–1.0)	0.8 (0.7–0.9)	0.8 (0.6–1.0)	0.143
Lactate, mmol/L	1.4 (1.0–1.8)	1.4 (1.0–1.7)	1.4 (1.0–1.8)	0.410
Severity of illness				
SOFA	5 (4,8)	5 (4,7)	7 (5,10)	< 0.001
SAPS II	36 (30–43)	35 (29–42)	41 (34–48)	< 0.001
On the first day after surgery				
Hemoglobin, g/dL	10.0 (9.1–11.1)	10.2 (9.2–11.2)	9.6 (8.8–10.6)	< 0.001
WBC, 10^9^/L	13.2 (10.6–16.4)	13.2 (10.6–16.2)	13.5 (10.5–17.2)	0.076
Platelet, 10^9^/L	147 (121–180)	148 (123–181)	140 (115–175)	< 0.001
Glucose, mg/dL	119 (106–134)	118 (106–133)	120.5 (105–139)	0.012
Serum creatinine, mg/dL	0.9 (0.8–1.1)	0.9 (0.8–1.1)	1.0 (0.8–1.3)	< 0.001
Lactate mean, mmol/L	2.0 (1.6–2.5)	1.9 (1.6–2.4)	2.3 (1.7–3.0)	< 0.001
Lactate maximum, mmol/L	2.6 (2.0–3.4)	2.5 (2.0–3.2)	3.1 (2.3–4.6)	< 0.001
In the first 2 days after surgery				
Use of vasoactive drugs	3941 (81.2%)	3174 (79.3%)	767 (89.7%)	< 0.001
Use of midazolam	229 (4.7%)	102 (2.6%)	127 (14.9%)	< 0.001
Use of IABP	164 (3.4%)	92 (2.3%)	72 (8.4%)	< 0.001
Use of RRT	129 (2.7%)	37 (0.9%)	92 (10.8%)	< 0.001
MV time, hours	8 (5–14)	8 (5–12)	16 (7–30)	< 0.001

*Note:* Values are expressed as median (interquartile range) or number of patients (%).

Abbreviations: ALT, alanine aminotransferase; AST, aspartate aminotransferase; BMI, body mass index; CABG, coronary artery bypass grafting; IABP, intra‐aortic balloon pump; MV, mechanical ventilation; NPOD, non‐postoperative delirium; POD, postoperative delirium; RRT, renal replacement therapy; SAPS II, simplified acute physiology score II; SOFA, sequential organ failure assessment; WBC, white blood cell.

### 3.2 PSM According to the Cut‐Off Value of Lactate‐Max

The cut‐off value of lactate‐max on the first postoperative day was determined based on the ROC curve, yielding 2.85 mmol/L (AUC 0.645; 95% CI 0.623–0.667). According to the lactate‐max, the patients were divided into lactate‐max < 2.85 mmol/L group and lactate‐max ≥ 2.85 mmol/L group. Patients in the lactate‐max ≥ 2.85 mmol/L group exhibited a greater prevalence of female patients and combined surgeries, an increased incidence of preoperative complications, a more extensive administration of postoperative vasoactive drugs, midazolam, intra‐aortic balloon pump, and renal replacement therapy, as well as prolonged mechanical ventilation duration. A 1:1 PSM was performed between the two groups, and the matching variables are detailed in Table 2. It was noted that 1276 patients were matched in each group, and the variables of the two groups were well‐balanced after PSM (SMD < 0.05) (Table 2, Figure S1).

**TABLE 2:** Baseline characteristics and clinical data of the two groups with different maximum lactate levels on the first postoperative day before and after PSM.

	Before PSM			After PSM		
	Lactate‐max < 2.85 mmol/L (*n* = 2859)	Lactate‐max ≥ 2.85 mmol/L (*n* = 1997)	SMD	Lactate‐max < 2.85 mmol/L (*n* = 1276)	Lactate‐max ≥ 2.85 mmol/L (*n* = 1276)	SMD
Baseline characteristics						
Age, years	67 (59–74)	68 (61–76)	0.127	68 (61–75)	68 (60–75.5)	0.020
Gender						
Male	2222 (77.7%)	1290 (64.6%)	0.293	865 (67.8%)	875 (68.6%)	0.017
Female	637 (22.3%)	707 (35.4%)		411 (32.2%)	401 (31.4%)	
Ethnicity						
White	1977 (69.2%)	1307 (65.5%)	0.078	852 (66.8%)	845 (66.2%)	0.010
African American	93 (3.2%)	70 (3.5%)		42 (3.3%)	44 (3.5%)	
Unknown/Others	789 (27.6%)	620 (31.0%)		382 (29.9%)	387 (30.3%)	
BMI, kg/m^2^	29.1 (25.9–32.7)	29.0 (25.6–33.3)	0.003	29.3 (25.8–32.9)	29.1 (25.7–33.4)	0.007
Admission type						
Elective	1236 (43.2%)	813 (40.7%)	0.039	545 (42.7%)	547 (42.9%)	0.003
Non‐elective	1623 (56.8%)	1184 (59.3%)		731 (57.3%)	729 (57.1%)	
Surgery type						
CABG only	1843 (64.5%)	1038 (52.0%)	0.293	692 (54.2%)	723 (56.7%)	0.021
Valve only	693 (24.2%)	558 (27.9%)		379 (29.7%)	337 (26.4%)	
Combined (CABG + valve)	323 (11.3%)	401 (20.1%)		205 (16.1%)	216 (16.9%)	
Concomitant disorders						
Congestive heart failure	637 (22.3%)	722 (36.2%)	0.313	395 (31.0%)	397 (31.1%)	0.003
Hypertension	1712 (59.9%)	1104 (55.3%)	0.086	732 (57.4%)	743 (58.2%)	0.017
Pulmonary hypertension	77 (2.7%)	140 (7.0%)	0.208	54 (4.2%)	48 (3.8%)	0.022
Chronic pulmonary disease	550 (19.2%)	443 (22.2%)	0.087	274 (21.5%)	267 (20.9%)	0.013
Peripheral vascular disease	343 (12.0%)	366 (18.3%)	0.161	201 (15.8%)	188 (14.7%)	0.029
Diabetes	1031 (36.1%)	801 (40.1%)	0.073	502 (39.3%)	488 (38.2%)	0.023
Chronic liver disease	88 (3.1%)	109 (5.5%)	0.125	47 (3.7%)	55 (4.3%)	0.031
Chronic kidney disease	465 (16.3%)	420 (21.0%)	0.105	250 (19.6%)	241 (18.9%)	0.018
Cerebrovascular disease	272 (9.5%)	214 (10.7%)	0.041	124 (9.7%)	135 (10.6%)	0.028
Dementia	10 (0.4%)	16 (0.8%)	0.041	6 (0.5%)	5 (0.4%)	0.011
Malignancy	78 (2.7%)	54 (2.7%)	0.012	36 (2.8%)	38 (3.0%)	0.010
Alcohol abuse	40 (1.4%)	22 (1.1%)	0.025	16 (1.3%)	16 (1.3%)	< 0.001
Mental disorder						
Anxiety	332 (11.6%)	252 (12.6%)	0.040	161 (12.6%)	154 (12.1%)	0.017
Depression	336 (11.8%)	233 (11.7%)	0.007	163 (12.8%)	149 (11.7%)	0.034
Preoperative data						
Hemoglobin, g/dL	12.4 (10.7–13.7)	12.0 (10.3–13.5)	0.136	12.1 (10.6–13.5)	12.2 (10.4–13.6)	0.020
WBC, 10^9^/L	9 (6.8–12.3)	9.6 (7.3–13.5)	0.135	9.3 (6.9–12.6)	9.5 (7.2–13.3)	0.022
Platelet, 10^9^/L	169 (131–215)	171 (128–221)	0.020	169 (130–217)	171 (129–217)	0.019
ALT, U/L	22 (15–31)	22 (15–33)	0.047	21 (15–31)	22 (16–33)	0.002
AST, U/L	24 (19–32)	25 (19–39)	0.055	24 (19–33)	24 (19–36)	0.006
Albumin, mg/dL	4.1 (3.8–4.4)	4.0 (3.7–4.3)	0.071	4.1 (3.7–4.4)	4.0 (3.7–4.4)	0.004
Glucose, mg/dL	110 (95–141)	115 (98–149)	0.105	111 (95–145)	113 (97–144)	0.017
Serum creatinine, mg/dL	0.8 (0.7–0.9)	0.8 (0.6–1.0)	0.035	0.8 (0.6–1.0)	0.8 (0.6–1.0)	0.008
Lactate, mmol/L	1.3 (1.0–1.6)	1.5 (1.2–2.0)	0.548	1.4 (1.1–1.8)	1.4 (1.1–1.8)	0.028
Severity of illness						
SOFA	5 (4–7)	6 (4–9)	0.517	6 (4–8)	6 (4–8)	0.013
SAPS II	34 (29–41)	38 (31–46)	0.285	36 (30–43)	36 (30–44)	0.002
On the first day after surgery						
Hemoglobin, g/dL	10.3 (9.3–11.3)	9.8 (9.0–10.8)	0.286	9.9 (9.0–11.0)	9.9 (9.1–10.9)	0.024
WBC, 10^9^/L	12.8 (10.4–15.8)	14.0 (11.0–17.6)	0.192	13.2 (10.8–16.2)	13.7 (10.8–17.1)	0.044
Platelet, 10^9^/L	148.5 (124–181)	145 (117–178)	0.064	146 (121–180)	146 (119–180)	0.017
Glucose, mg/dL	117 (105–132)	121 (107–138)	0.151	119 (106–133)	119 (105–135)	0.024
Serum creatinine, mg/dL	0.9 (0.8–1.0)	1.0 (0.8–1.2)	0.066	0.9 (0.7–1.2)	0.9 (0.8–1.2)	0.002
In the first 2 days after surgery						
Use of vasoactive drugs	2204 (77.1%)	1737 (87.0%)	0.233	1071 (83.9%)	1062 (83.2%)	0.019
Use of midazolam	74 (2.6%)	155 (7.8%)	0.204	45 (3.5%)	50 (3.9%)	0.018
Use of IABP	56 (2.0%)	108 (5.4%)	0.163	18 (1.4%)	15 (1.2%)	0.016
Use of RRT	27 (0.9%)	102 (5.1%)	0.209	23 (1.8%)	21 (1.7%)	0.010
MV time, hours	8 (5–11)	10 (6–22)	0.319	8 (5–12)	9 (5–18)	0.021

*Note:* Values are expressed as median (interquartile range) or number of patients (%).

Abbreviations: ALT, alanine aminotransferase; AST, aspartate aminotransferase; BMI, body mass index; CABG, coronary artery bypass grafting; IABP, intra‐aortic balloon pump; MV, mechanical ventilation; PSM, propensity score matching; RRT, renal replacement therapy; SAPS II, simplified acute physiology score II; SMD, standard mean difference; SOFA, sequential organ failure assessment; WBC, white blood cell.

### 3.3 Outcomes of the Two Groups With Different Lactate‐Max Levels Before and After PSM

As shown in Table 3, before PSM, the incidence of POD (*p* < 0.001), in‐hospital mortality (*p* < 0.001), and 30‐day mortality (*p* < 0.001) were markedly elevated in the lactate‐max ≥ 2.85 mmol/L group compared with the lactate‐max < 2.85 mmol/L group, and the duration of ICU stay (*p* < 0.001) and hospital stay (*p* < 0.001) of the lactate‐max ≥ 2.85 mmol/L group were significantly prolonged. After PSM, the incidence of POD (*p* = 0.029) in the lactate‐max ≥ 2.85 mmol/L group was still considerably higher, and the duration of ICU stay (*p* < 0.001) and hospital stay (*p* = 0.006) were markedly prolonged. However, the two groups had no significant differences in in‐hospital mortality (*p* = 1) and 30‐day mortality (*p* = 0.713).

**TABLE 3:** Outcomes of the two groups with different maximum lactate levels on the first postoperative day before and after PSM.

	Lactate‐max < 2.85 mmol/L (*n* = 2859)	Lactate‐max ≥ 2.85 mmol/L (*n* = 1997)	*p*	Lactate‐max < 2.85 mmol/L (*n* = 1276)	Lactate‐max ≥ 2.85 mmol/L (*n* = 1276)	*p*
Outcomes						
Delirium	353 (12.4%)	502 (25.1%)	< 0.001	203 (15.9%)	245 (19.2%)	0.029
LOIS, days	1.5 (1.2–2.5)	2.3 (1.3–4.2)	< 0.001	2.0 (1.3–3.2)	2.2 (1.3–3.3)	< 0.001
LOHS, days	7.0 (5.3–9.9)	8.1 (5.9–12.8)	< 0.001	7.3 (5.5–10.7)	7.7 (5.7–11.5)	0.006
In‐hospital mortality	12 (0.4%)	43 (2.2%)	< 0.001	8 (0.6%)	8 (0.6%)	1.000
30‐day mortality	21 (0.7%)	63 (3.2%)	< 0.001	14 (1.1%)	16 (1.3%)	0.713

Abbreviations: LOHS, length of hospital stay; LOIS, length of intensive care unit stay; PSM, propensity score matching.

### 3.4 Association Between Different Lactate‐Max Levels and POD

According to the cut‐off value of lactate‐max on the first postoperative day of 2.85 mmol/L, the lactate‐max was transformed from a continuous variable into a binary variable, namely lactate‐max < 2.85 mmol/L or lactate‐max ≥ 2.85 mmol/L. The correlation between the lactate‐max levels and POD was evaluated using the univariate logistic regression, multivariate logistic regression, PSM, and IPTW models, respectively. The findings indicated a substantial correlation between the lactate‐max and POD across the four models (Table 4). The variables included in the multivariate logistic regression model are presented in Table S2. The AUC of the ROC curve derived from the multivariate model was 0.769 (95% CI 0.751–0.787).

**TABLE 4:** Association between the maximum lactate levels on the first postoperative day and POD.

Model	OR	95% CI	*p*
Unadjusted	2.38	2.05–2.77	< 0.001
Multivariable	1.37	1.15–1.62	< 0.001
PSM	1.27	1.02–1.59	0.036
IPTW	1.30	1.09–1.55	0.004

Abbreviations: CI, confidence interval; IPTW, inverse probability of treatment weighting; OR, odds ratio; POD, postoperative delirium; PSM, propensity score matching.

**FIGURE 2:**
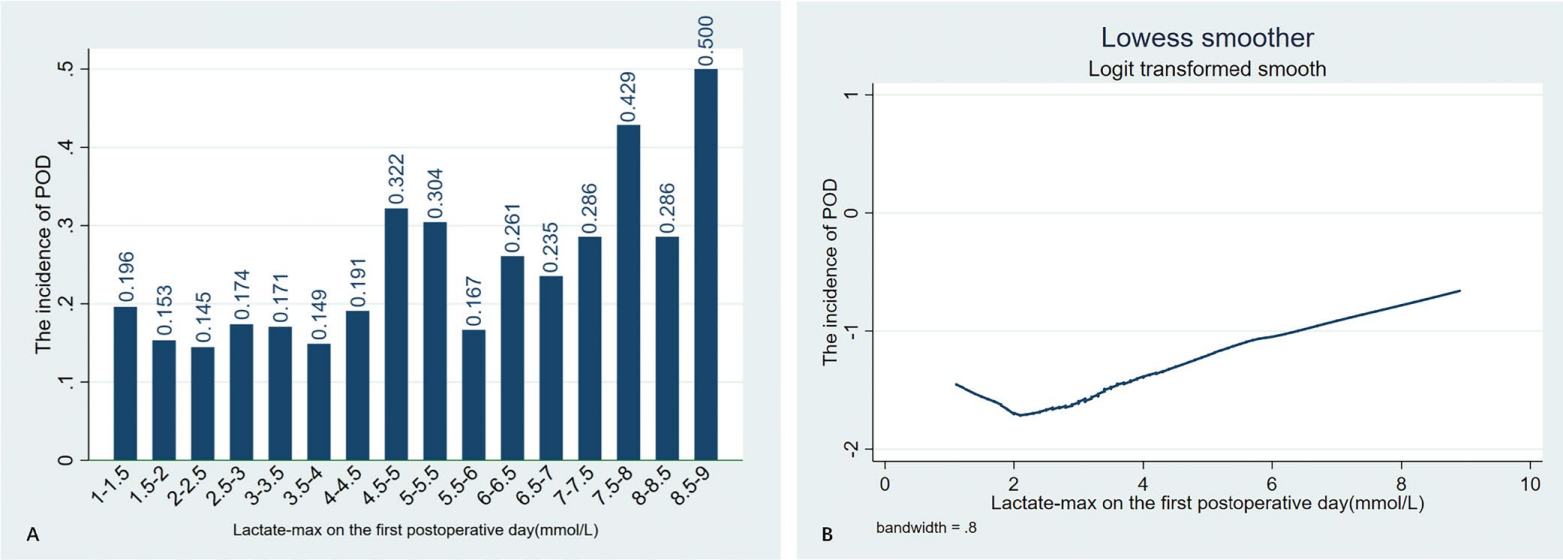
Bar graph (Figure 2A) and Lowess Smoother (Figure 2B) between the lactate maximum on the first postoperative day and the incidence of POD. POD, postoperative delirium.

Furthermore, we employed a bar graph and the Lowess Smoothing method to illustrate the correlation between the first postoperative day lactate‐max (1–9 mmol/L) and the incidence of POD (Figure 2). The Lowess smoother revealed a ‘U’‐shaped nonlinear relationship between the lactate‐max and the incidence of POD, with the threshold of lactate‐max of around 2.4 mmol/L.

### 3.5 Association Between Dynamic Changes in Postoperative Lactate Levels and POD

We examined the dynamic changes in postoperative lactate levels in both patients without POD and patients with POD during the initial 12 h following ICU admission by dividing the 12 h into four equal intervals. As depicted in Figure S2, lactate levels in patients with POD were significantly higher than those in patients without POD across all four time periods: 0–3 h (T_1_), 3–6 h (T_2_), 6–9 h (T_3_), and 9–12 h (T_4_) after surgery (Table S3). Additionally, through univariate logistic regression analysis, we determined that elevated lactate levels at T_1_, T_2_, T_3_, and T_4_ were all independent risk factors for POD (Table S4).

### 3.6 Subgroup Analyses and Interaction Analyses Between Stratification Variables and Lactate‐Max

We conducted subgroup analyses among the PSM patients. It can be seen in Table S5 that there was no interaction between stratification variables, including gender, admission type, history of cerebrovascular disease, early postoperative administration of midazolam and postoperative mechanical ventilation duration, and lactate‐max. This indicates that the correlation between lactate‐max and the incidence of POD remained consistent across the five subgroups. However, there was an interaction between age and lactate‐max on the first postoperative day (*p* for interaction = 0.019). In patients over 65 years of age, elevated lactate‐max showed a significant association with an increased incidence of POD.

## 4 Discussion

### 4.1 The Current State of Research and the Findings of This Study on Lactate and POD

The findings of this study suggested that the elevated lactate‐max on the first postoperative day was associated with an increased risk of POD. The cutoff value of lactate‐max on the first postoperative day was 2.85 mmol/L. The incidence of POD in patients with a lactate‐max ≥ 2.85 mmol/L was significantly higher, and both the duration of ICU stay and hospital stay were significantly extended compared to those with a lactate‐max < 2.85 mmol/L. Furthermore, subgroup analysis revealed an interaction between the maximum lactate levels on the first postoperative day and age, and the findings of this study are more relevant to patients over 65 years old.

Although the AUC for the ROC curve between lactate‐max and POD was 0.645, the AUC of the multivariate model that incorporated lactate‐max increased to 0.769. Furthermore, based on the cut‐off value derived from the ROC curve, patients were categorized into two groups, and the results demonstrated a significant difference in POD incidence between the two groups. As supported by previous literature and clinical experience, the occurrence of POD is influenced by multiple factors, making it challenging to accurately predict POD using only a single variable. This complexity likely explains why the AUC for the ROC curve between lactate‐max and POD was somewhat low in this study.

A prospective observational study with small samples conducted in Nanjing Drum Tower Hospital, China, revealed that postoperative lactate level was an independent predictor of POD in patients undergoing cardiac surgery [26]. A retrospective cohort study in elderly trauma patients indicated that lactate levels 1 h after surgery were predictive for POD [27]. However, there was no association between serum lactate at ICU admission and neurological disorders in a retrospective study involving patients undergoing elective neurosurgeries [28].

As can be seen, the correlation between postoperative lactate and POD has received increasing attention recently, but the results were not completely consistent, potentially due to the small samples and study design. Therefore, using the data from real patients in the United States, we conducted this large‐sample study to explore the relationship between the highest lactate level on the first postoperative day and POD in patients following cardiac surgery.

### 4.2 Potential Mechanisms Underlying the Association Between Lactate and POD

Recent research has shown that lactate is not only a vital physiological metabolite, but also an essential multifunctional metabolic fuel in cellular bioenergetics [29, 30]. The brain lacks energy reserves, relying primarily on circulating substrates such as glucose, lactate, ketones, and glutamate for its energy supply [31]. The well‐known hypothesis of the astrocyte‐neuron lactate shuttle (ANLS) posits that with the activity of neurons, the energy consumption of neurons increases, the Na^+^‐dependent transporters and Na^+^/K^+^ adenosine triphosphatase of glial cells are activated, and the glycolysis and extracellular lactate of glial cells increase. With the enhancement in neuronal excitability, there is a decline in neuronal glucose consumption and an increase in lactate utilization, while the glycolytic activity of glial cells supplies lactate reserves [32, 33, 34].

Therefore, as an essential substrate of cerebral metabolism, extracellular lactate in the brain is a vital indicator of the cerebral metabolic state. When the brain's energy consumption and metabolic equilibrium are disrupted, the concentrations of lactate and glucose in the brain fluctuate, leading to a condition of hypoglycemia and elevated lactate, known as an acute metabolic crisis (ACMC) [28, 35]. ACMC is a well‐recognized reason for secondary brain injury and can result in acute neurological dysfunction [35, 36]. However, the assessment of cerebral extracellular lactate concentrations requires an intra‐cerebral micro‐dialysis catheter, an invasive and costly procedure that poses challenges for widespread clinical implementation. Simultaneously, the elevation in serum lactate is regarded as indicative of a corresponding rise in cerebral extracellular lactate to a certain degree [28].

Based on the discussion above, we conclude that the elevated serum lactate levels in the early postoperative period may, to some extent, reflect an increase in cerebral extracellular lactate. Cerebral extracellular lactate serves as a crucial substrate for cerebral metabolism. When the concentration of cerebral extracellular lactate increases, it suggests that the energy consumption and metabolic balance within the brain may be disrupted, potentially leading to the occurrence of ACMC, which could result in acute neurological dysfunction, such as POD.

### 4.3 Implications of the Findings for Clinical Practice

Lactate elevation can arise from a multitude of causes. Therefore, the guiding principle with clinical recommendations for reducing lactate is to consider the potential factors contributing to the elevated lactate and subsequently implement corresponding treatments, including intravenous fluid therapy, blood product supplementation, adjustment of oxygen supply, the administration of vasopressors and appropriate positive inotropic agents, strict blood glucose control, and discontinuing the use of pharmacological agents that may induce hyperlactatemia [37, 38, 39]. Based on current evidence, specific pharmacological interventions aimed at accelerating lactate metabolism or inhibiting lactate production are not recommended. Moreover, the administration of buffers such as sodium bicarbonate for treating acidosis remains a controversial topic [37].

Although previous research has indicated that lactate reduction can contribute to the decrease in morbidity and mortality among critically ill patients [40, 41], few studies have explored the impact of lactate reduction on POD in surgical patients. A retrospective study demonstrated that the decline in lactate levels within 24 h of ICU admission was associated with a reduced incidence of delirium in elderly critically ill patients, but the specific strategies for reducing lactate were not elaborated on [42]. Further studies are needed to directly investigate the effects of various lactate reduction measures on POD incidence.

### 4.4 Limitations

First of all, the outcomes of this investigation were derived from a single‐center cohort study, which constrained the generalizability of the conclusions. Secondly, the absence of data on some key variables that may affect lactate levels and POD, such as patients' educational attainment, preoperative cognitive function evaluation, intraoperative CPB duration and lactate levels, and postoperative pain scores in the MIMIC‐IV database, meant we could not account for these critical confounding variables in our models to refine the analysis of the dynamic changes in lactate. Furthermore, the reduction in sample size from 4856 to 2552 after PSM may result in reduced statistical power, thereby increasing the risk of type II error. To mitigate this issue, we employed 1:1 matching and adjusted the caliper of the matching. Although the sample size was reduced, the balance of the sample was improved after matching, which helped to reduce the influence of confounding factors and improve the validity of the results. Additionally, we used the IPTW model to evaluate the relationship between postoperative lactate levels and POD while retaining the sample size, and the results were consistent. Moreover, according to the causes of lactate elevation, hyperlactatemia is classified into type A and type B. However, in this study, as we failed to obtain additional parameters such as mixed venous oxygen partial pressure, mixed venous blood oxygen saturation, and oxygen uptake rate to evaluate the adequacy of oxygen delivery when measuring lactate levels, we were unable to accurately determine the type of lactate elevation, which limited our in‐depth understanding of the results. Finally, the study did not include information on the type, duration, and severity of delirium, which may restrict our comprehensive understanding of the relationship between lactate‐max on the first postoperative day and POD.

## 5 Conclusion

In conclusion, our findings suggest that the first postoperative day lactate‐max was associated with the risk of POD in patients undergoing cardiac surgery, with the POD risk increasing significantly in patients with a lactate‐max ≥ 2.85 mmol/L on the first postoperative day. In the future, further research is needed to ascertain whether evaluation and treatment based on the first postoperative day lactate‐max can reduce the incidence of POD effectively and elucidate its underlying mechanisms.

## Author Contributions

R.A. and X.W. conceived the idea and interpreted the results. R.A., D.B., and X.W. extracted and collected the data. R.A., Y.L., and J.D. analyzed the data. R.A., X.W., and D.B. drafted the manuscript. F.Y., Y.J., and S.Y. revised the manuscript. All the authors read and approved the final manuscript.

## Ethics Statement

The Institutional Review Boards of the Massachusetts Institute of Technology (Cambridge, MA, USA) and Beth Israel Deaconess Medical Center (Boston, MA, USA) approved the use of the MIMIC‐IV database for the present study (Record ID: 55754432).

## Consent

Data extracted from the MIMIC‐IV database do not require individual informed consent because the research data are publicly available, and all patient data are de‐identified.

## Conflicts of Interest

The authors declare no conflicts of interest.

## Supporting information


**Figure S1.** Equilibrium test of propensity score matching. SOFA, sequential organ failure assessment; MV, mechanical ventilation; SAPS II, simplified acute physiology score II; RRT, renal replacement therapy; WBC, white blood cell; IABP, intra‐aortic balloon pump; AST, aspartate aminotransferase; ALT, alanine aminotransferase; BMI, body mass index.

**Figure S2.** Postoperative dynamic changes in lactate levels in patients from the NPOD and POD groups. T1, within 3 h after admission to the ICU; T2, from 3 to 6 h after admission to the ICU; T3, from 6 to 9 h after admission to the ICU; T4, from 9 to 12 h after admission to the ICU. NPOD, non‐postoperative delirium; POD, postoperative delirium ICU, intensive care unit.

Appendix S1.

## Data Availability

The data supporting the findings of this study are openly available in the MIMIC‐IV database (v 2.2) at https://physionet.org/content/mimiciv/2.2/.
