# Supplementary figures and images for: Association Between the Highest Lactate Level on the First Postoperative Day and Postoperative Delirium in Cardiac Surgery Patients
Source: CNS Neurosci Ther. 2025 Apr 22;31(4):e70380. doi: 10.1111/cns.70380 (PMC12012567; doi:10.1111/cns.70380)

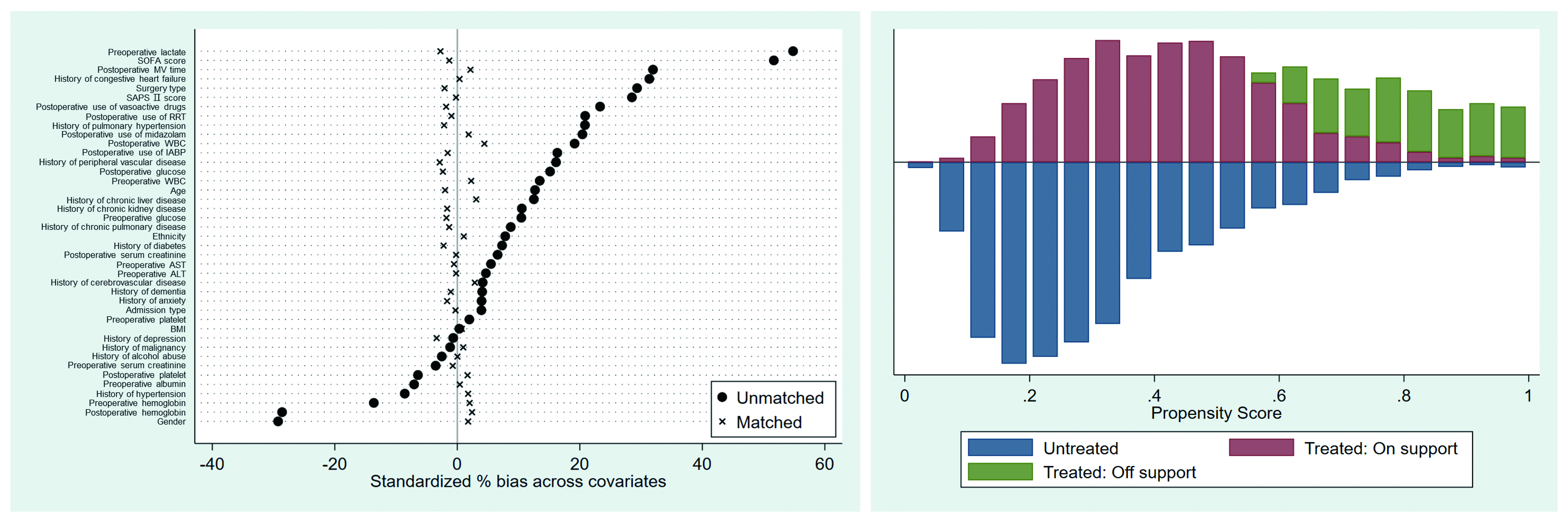

Supplement: Supplementary file 1 — Figure S1. Equilibrium test of propensity score matching. SOFA, sequential organ failure assessment; MV, mechanical ventilation; SAPS II, simplified acute physiology score II; RRT, renal replacement therapy; WBC, white blood cell; IABP, intra‐aortic balloon pump; AST, aspartate aminotransferase; ALT, alanine aminotransferase; BMI, body mass index. [file CNS-31-e70380-s002.tif]

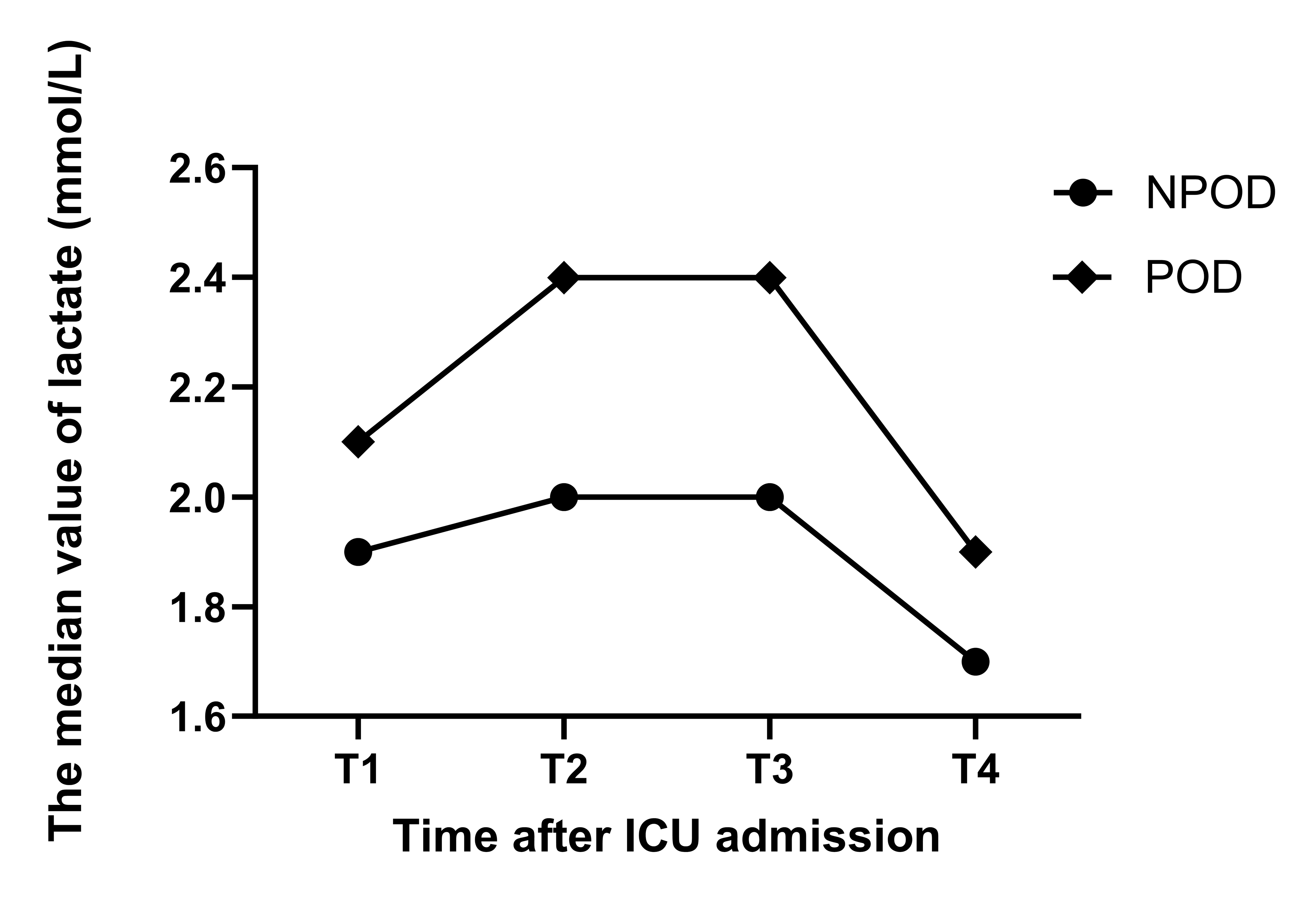

Supplement: Supplementary file 2 — Figure S2. Postoperative dynamic changes in lactate levels in patients from the NPOD and POD groups. T1, within 3 h after admission to the ICU; T2, from 3 to 6 h after admission to the ICU; T3, from 6 to 9 h after admission to the ICU; T4, from 9 to 12 h after admission to the ICU. NPOD, non‐postoperative delirium; POD, postoperative delirium ICU, intensive care unit. [file CNS-31-e70380-s001.tif]
