# Supplementary material for: Association Between the Highest Lactate Level on the First Postoperative Day and Postoperative Delirium in Cardiac Surgery Patients
Source: CNS Neurosci Ther. 2025 Apr 22;31(4):e70380. doi: 10.1111/cns.70380 (PMC12012567; doi:10.1111/cns.70380)
Supplement: Supplementary file 3 — Appendix S1. [file CNS-31-e70380-s003.docx]

**Table S1. ICD codes for identifying CABG and valve surgery.**

| Surgery type | ICD version | ICD codes |
| --- | --- | --- |
| CABG | ICD-9 | 3610, 3611, 3612, 3613, 3614, 3615, 3616, 3617 |
|  | ICD-10 | 210083, 210088, 210089, 021008C, 021008F, 021008W, 210093, 210098, 210099, 021009C, 021009F, 021009W, 02100A3, 02100A8, 02100A9, 02100AC, 02100AF, 02100AW, 02100J3, 02100J8, 02100J9, 02100JC, 02100JF, 02100JW, 02100K3, 02100K8, 02100K9, 02100KC, 02100KF, 02100KW, 02100Z3, 02100Z8, 02100Z9, 02100ZC, 02100ZF, 211083, 211088, 211089, 021108C, 021108F, 021108W, 211093, 211098, 211099, 021109C, 021109F, 021109W, 02110A3, 02110A8, 02110A9, 02110AC, 02110AF, 02110AW, 02110J3, 02110J8, 02110J9, 02110JC, 02110JF, 02110JW, 02110K3, 02110K8, 02110K9, 02110KC, 02110KF, 02110KW, 02110Z3, 02110Z8, 02110Z9, 02110ZC, 02110ZF, 212083, 212088, 212089, 021208C, 021208F, 021208W, 212093, 212098, 212099, 021209C, 021209F, 021209W, 02120A3, 02120A8, 02120A9, 02120AC, 02120AF, 02120AW, 02120J3, 02120J8, 02120J9, 02120JC, 02120JF, 02120JW, 02120K3, 02120K8, 02120K9, 02120KC, 02120KF, 02120KW, 02120Z3, 02120Z8, 02120Z9, 02120ZC, 02120ZF, 213083, 213088, 213089, 021308C, 021308F, 021308W, 213093, 213098, 213099, 021309C, 021309F, 021309W, 02130A3, 02130A8, 02130A9, 02130AC, 02130AF, 02130AW, 02130J3, 02130J8, 02130J9, 02130JC, 02130JF, 02130JW, 02130K3, 02130K8, 02130K9, 02130KC, 02130KF, 02130KW, 02130Z3, 02130Z8, 02130Z9, 02130ZC, 02130ZF |
| Valve surgery | ICD-9 | 3520, 3521, 3522, 3523, 3524, 3525, 3526, 3527, 3528 |
|  | ICD-10 | 02QF0ZJ, 02QF0ZZ, 02QG0ZE, 02QG0ZZ, 02QH0ZZ, 02QJ0ZG, 02QJ0ZZ, 02RF07Z, 02RF08Z, 02RF0JZ, 02RF0KZ, 02RG07Z, 02RG08Z, 02RG0JZ, 02RG0KZ, 02RH07Z, 02RH08Z, 02RH0JZ, 02RH0KZ, 02RJ07Z, 02RJ08Z, 02RJ0JZ, 02RJ0KZ |

Abbreviation: ICD, International Classification of Diseases; CABG, coronary artery bypass grafting.

**Table S2. Univariate and multivariate logistic regression analyses between baseline characteristics and clinical data and POD.**

|  | **Univariate logistic regression** | | | **Multivariate logistic regression** | | |
| --- | --- | --- | --- | --- | --- | --- |
|  | **OR** | **95% CI** | ***p* value** | **OR** | **95% CI** | ***p* value** |
| **Baseline characteristics** |  |  |  |  |  |  |
| Age, years | 1.02 | 1.01-1.03 | 0.001 |  |  |  |
| Gender | 1.02 | 0.82-1.26 | 0.875 |  |  |  |
| Male |  |  |  |  |  |  |
| Female |  |  |  |  |  |  |
| Ethnicity | 0.95 | 0.86-1.05 | 0.299 |  |  |  |
| White |  |  |  |  |  |  |
| African American |  |  |  |  |  |  |
| Unknown/Others |  |  |  |  |  |  |
| BMI, kg/m^2^ | 0.98 | 0.97-0.99 | 0.027 |  |  |  |
| Admission type | 1.25 | 1.06-1.49 | 0.009 | 1.21 | 1.02-1.43 | 0.032 |
| Elective |  |  |  |  |  |  |
| Non-elective |  |  |  |  |  |  |
| Surgery type | 1.40 | 1.26-1.56 | <0.001 | 1.34 | 1.20-1.50 | <0.001 |
| CABG only |  |  |  |  |  |  |
| Valve only |  |  |  |  |  |  |
| Combined (CABG + valve) |  |  |  |  |  |  |
| **Concomitant disorders** |  |  |  |  |  |  |
| Congestive heart failure | 0.99 | 0.80-1.23 | 0.936 |  |  |  |
| Hypertension | 0.97 | 077-1.22 | 0.796 |  |  |  |
| Pulmonary hypertension | 1.12 | 0.77-1.64 | 0.555 |  |  |  |
| Chronic pulmonary disease | 0.98 | 0.79-1.21 | 0.835 |  |  |  |
| Peripheral vascular disease | 1.37 | 1.11-1.70 | 0.003 | 1.32 | 1.07-1.64 | 0.011 |
| Diabetes | 1.50 | 1.26-1.78 | <0.001 | 1.44 | 1.22-1.71 | <0.001 |
| Chronic liver disease | 0.63 | 0.39-1.01 | 0.054 |  |  |  |
| Chronic kidney disease | 1.26 | 1.02-1.54 | 0.029 |  |  |  |
| Cerebrovascular disease | 1.43 | 1.12-1.82 | 0.002 | 1.41 | 1.10-1.80 | 0.006 |
| Dementia | 1.79 | 0.68-4.68 | 0.235 |  |  |  |
| Malignancy | 1.05 | 0.62-1.78 | 0.845 |  |  |  |
| Alcohol abuse | 1.79 | 0.90-3.56 | 0.097 |  |  |  |
| Mental disorder |  |  |  |  |  |  |
| Anxiety | 1.28 | 0.97-1.69 | 0.080 |  |  |  |
| Depression | 1.43 | 1.13-1.80 | 0.003 | 1.44 | 1.14-1.82 | 0.002 |
| **Preoperative data** |  |  |  |  |  |  |
| Hemoglobin, g/dL | 0.96 | 0.91-1.01 | 0.156 |  |  |  |
| WBC, 10^9^/L | 1.02 | 1.00-1.05 | 0.082 |  |  |  |
| Platelet, 10^9^/L | 1.00 | 1.00-1.00 | 0.511 |  |  |  |
| ALT, U/L | 1.00 | 1.00-1.00 | 0.602 |  |  |  |
| AST, U/L | 1.00 | 1.00-1.00 | 0.418 |  |  |  |
| Albumin, mg/dL | 0.89 | 0.73-1.09 | 0.273 |  |  |  |
| Glucose, mg/dL | 1.00 | 1.00-1.00 | 0.863 |  |  |  |
| Serum creatinine, mg/dL | 0.50 | 0.33-0.74 | 0.001 |  |  |  |
| Lactate, mmol/L | 0.85 | 0.74-0.99 | 0.035 |  |  |  |
| **Severity of illness** |  |  |  |  |  |  |
| SOFA | 1.12 | 1.07-1.18 | <0.001 | 1.13 | 1.10-1.17 | <0.001 |
| SAPS II | 1.00 | 0.99-1.01 | 0.550 |  |  |  |
| **On the first day after surgery** |  |  |  |  |  |  |
| Hemoglobin, g/dL | 0.91 | 0.83-0.99 | 0.025 |  |  |  |
| WBC, 10^9^/L | 0.99 | 0.97-1.01 | 0.261 |  |  |  |
| Platelet, 10^9^/L | 1.00 | 1.00-1.00 | 0.347 |  |  |  |
| Glucose, mg/dL | 1.00 | 1.00-1.00 | 0.608 |  |  |  |
| Serum creatinine, mg/dL | 1.49 | 1.14-1.94 | 0.004 |  |  |  |
| Lactate maximum, mmol/L | 1.53 | 1.30-1.81 | <0.001 | 1.37 | 1.15-1.62 | <0.001 |
| **In the first 2 days after surgery** |  |  |  |  |  |  |
| Use of vasoactive drugs | 1.26 | 0.95-1.55 | 0.105 |  |  |  |
| Use of midazolam | 2.18 | 1.54-3.09 | <0.001 | 1.98 | 1.39-2.80 | <0.001 |
| Use of IABP | 2.13 | 1.44-3.14 | 0.001 | 1.95 | 1.32-2.89 | 0.001 |
| Use of RRT | 3.32 | 2.07-5.34 | <0.001 | 2.31 | 1.43-3.72 | 0.001 |
| MV time, hours | 1.02 | 1.01-1.03 | <0.001 | 1.02 | 1.01-1.02 | <0.001 |

Abbreviations: POD, postoperative delirium; OR, odds ratio; CI, confidence interval; BMI, body mass index; CABG, coronary artery bypass grafting; WBC, white blood cell; ALT, alanine aminotransferase; AST, aspartate aminotransferase; SOFA, sequential organ failure assessment; SAPS Ⅱ, simplified acute physiology score Ⅱ; IABP, intra-aortic balloon pump; RRT, renal replacement therapy; MV, mechanical ventilation.

**Table S3. The dynamic changes of postoperative lactate in NPOD and POD groups.**

| **Variables** | **NPOD (n=4001)** | **POD (n=855)** | ***p* value** |
| --- | --- | --- | --- |
| T_1_ lactate (mmol/L) | 1.9 (1.4-2.5) | 2.1 (1.5-2.9) | <0.001 |
| T_2_ lactate (mmol/L) | 2.0 (1.5-2.7) | 2.4 (1.7-3.4) | <0.001 |
| T_3_ lactate (mmol/L) | 2.0 (1.5-2.8) | 2.4 (1.7-3.8) | <0.001 |
| T_4_ lactate (mmol/L) | 1.7 (1.3-2.4) | 1.9 (1.3-2.8) | 0.001 |

*Note*: T_1_ lactate, lactate within 3 hours after admission to ICU; T_2_ lactate, lactate from 3 to 6 hours after admission to the ICU; T_3_ lactate, lactate from 6 to 9 hours after admission to the ICU; T_4_ lactate, lactate from 9 to 12 hours after admission to the ICU.

Abbreviations: NPOD, non-postoperative delirium; POD, postoperative delirium.

**Table S4. Association between dynamic changes in lactate on the first postoperative day and POD.**

| **Variables** | **OR** | **95%CI** | ***p* value** |
| --- | --- | --- | --- |
| T_1_ lactate (mmol/L) | 1.38 | 1.29-1.48 | <0.001 |
| T_2_ lactate (mmol/L) | 1.42 | 1.32-1.52 | <0.001 |
| T_3_ lactate (mmol/L) | 1.30 | 1.22-1.38 | <0.001 |
| T_4_ lactate (mmol/L) | 1.25 | 1.15-1.35 | <0.001 |

*Note*: T_1_ lactate, lactate within 3 hours after admission to ICU; T_2_ lactate, lactate from 3 to 6 hours after admission to the ICU; T_3_ lactate, lactate from 6 to 9 hours after admission to the ICU; T_4_ lactate, lactate from 9 to 12 hours after admission to the ICU.

Abbreviations: POD, postoperative delirium; OR, odds ratio; CI, confidence interval.

**Table S5. Subgroup analyses and interaction analyses between stratification variables and maximum lactate levels on the first postoperative day.**

| **Subgroup analyses** | **N (PSM)** | **OR (95%CI)** | ***p* for interaction** |
| --- | --- | --- | --- |
| Age, years |  |  |  |
| ≤ 65 | 1018 (39.9%) | 1.04 (0.70-1.55) | 0.009 |
| > 65 | 1534 (60.1%) | 1.38 (1.05-1.82) |  |
| Gender |  |  |  |
| Male | 1740 (68.2%) | 1.43 (1.07-1.90) | 0.069 |
| Female | 812 (31.8%) | 1.03 (0.71-1.50) |  |
| Admission type |  |  |  |
| Non-elective | 1092 (42.8%) | 1.20 (0.90-1.60) | 0.729 |
| Elective | 1460 (57.2%) | 1.31 (0.92-1.88) |  |
| History of cerebrovascular disease |  |  |  |
| Yes | 259 (10.1%) | 1.16 (0.60-2.23) | 0.726 |
| No | 2293 (89.9%) | 1.30 (1.02-1.65) |  |
| Postoperative use of midazolam |  |  |  |
| Yes | 95 (3.7%) | 1.33 (0.47-3.74) | 0.890 |
| No | 2457 (96.3%) | 1.25 (0.99-1.57) |  |
| MV time, hours |  |  |  |
| ≥ 24 | 298 (11.7%) | 0.93 (0.55-1.59) | 0.190 |
| < 24 | 2254 (88.3%) | 1.30 (1.01-1.66) |  |

Abbreviations: PSM, propensity score matching; OR, odds ratio; CI, confidence interval; MV, mechanical ventilation.

**Figure Legends**

**Figure S1. Equilibrium test of propensity score matching.**

Abbreviations: SOFA, sequential organ failure assessment; MV, mechanical ventilation; SAPS Ⅱ, simplified acute physiology score Ⅱ; RRT, renal replacement therapy; WBC, white blood cell; IABP, intra-aortic balloon pump; AST, aspartate aminotransferase; ALT, alanine aminotransferase; BMI, body mass index.

**Figure S2. Postoperative dynamic changes in lactate levels in patients from the NPOD and POD groups.**

*Note*: T1, within 3 hours after admission to the ICU; T2, from 3 to 6 hours after admission to the ICU; T3, from 6 to 9 hours after admission to the ICU; T4, from 9 to 12 hours after admission to the ICU.

Abbreviations: NPOD, non-postoperative delirium; POD, postoperative delirium ICU, intensive care unit.
